# Were economic evaluations well reported for the newly listed oncology drugs in China’s national reimbursement drug list

**DOI:** 10.1186/s12913-022-08858-7

**Published:** 2022-12-03

**Authors:** Liu Liu, Zhixin Jiang, Fuming Li, Yan Wei, Jian Ming, Yi Yang, Shimeng Liu, Lizheng Shi, Yingyao Chen

**Affiliations:** 1 School of Public Health, Fudan University, Shanghai, China; 2 NHC Key Laboratory of Health Technology Assessment (Fudan University), Shanghai, China; 3 Real World Solutions, IQVIA China, Shanghai, China; 4 School of Public Health and Tropical Medicine, Tulane University, New Orleans, LA USA

**Keywords:** China, Economic evaluation, Oncology drugs, Price negotiation, Reporting quality assessment

## Abstract

**Purpose:**

To assess the reporting quality of published economic evaluations of the negotiated oncology drugs listed for China’s 2020 National Reimbursement Drug List (NRDL).

**Methods:**

A comprehensive search was conducted to identify economic evaluation studies of negotiated oncology drugs listed in China’s 2020 NRDL using the PubMed/MEDLINE, Embase, Web of Science, CNKI, SinoMed, and WanFang Database up to March 31, 2021. The Consolidated Health Economic Evaluation Reporting Standards (CHEERS) checklist scored the reporting quality between 0 and 100. A linear regression analysis was employed to examine the influence of various characteristics on the reporting quality scores.

**Results:**

Eighty papers were included in the study, with the majority published during the past decade. Furthermore, more than half of the articles (57.5%, or 46 out of 80) were written in English. The average CHEERS score was 74.63 ± 12.75 and ranged from 43.48 to 93.75. The most inadequately reported items included choice of model, characterization of heterogeneity, and discussion, as well as currency, price date and conversion. Higher scores were associated with articles published from 2019 to 2021 and English publications.

**Conclusion:**

The economic evaluation studies of negotiated oncology drugs listed in 2020 NRDL had moderate reporting quality. The Chinese economic evaluation publications could improve the reporting quality if the CHEERS checklist is consistently implemented. Also, the Chinese journals maybe explore introducing a reporting standard for economic evaluations.

**Supplementary Information:**

The online version contains supplementary material available at 10.1186/s12913-022-08858-7.

## Introduction

With the growing demand for medical care, health spending and pharmaceutical expenditure has increased rapidly over the past few decades [1, 2]. Drug expenditure accounts for 30% of total health expenditure in China [3], far higher than that proportion in the Organization for Economic Co-operation and Development (OECD) countries [4]. Drug expenditure rose by 664% from 2001 to 2016 [5]. The Chinese government initiates price negotiations for drugs included in the reimbursement list to contain pharmaceutical expenditures and expand access to medicines. A pilot, national drug price negotiation, was launched in 2016 [6], and the National Reimbursement Drug List (NRDL) price negotiations have been formally organized by the health insurance department annually since 2017. As a result, five drug price negotiations were conducted in China by the end of 2020.

Cancer is one of China’s leading causes of death [7]. In 2019, approximately 2.717 million cancer deaths accounted for 25.48% of all-cause mortality [8]. Although innovative oncology drugs have greatly improved the treatment outcome and quality of life of patients, which significantly enhanced patient survival rates [9, 10], their high prices have led to poor patient affordability [11]. Therefore, to incorporate more oncology drugs with significant efficacy into the list and reduce the personal economic burden of insured cancer patients, oncology drugs have become the focus of the NRDL price negotiations.

In May 2016, the results of the first pilot drug price negotiation in China were announced, and this negotiation successfully negotiated three drugs, two of which are oncology drugs. The outcome of the NRDL price negotiations from 2017 to 2020 shows that eighteen negotiated oncology drugs were included in the list in 2017 [12], accounting for 50% of all negotiated drugs in the NRDL, and only oncology drugs were negotiated in 2018, with seventeen drugs listed [13]. Twenty-two and thirty-one listed oncology drugs, accounting for 22.7 and 26.1% of all negotiated drugs in the NRDL in 2019 and 2020, respectively [14, 15].

As an indispensable part of the NRDL price negotiation, economic evaluations are designed to inform decisions that estimate the cost and effectiveness trade-off of two or more drugs, programs, or interventions, which play a significant role in decision-making on insurance coverage and reimbursement prices [16]. Therefore, economic evaluation as an essential tool was introduced in negotiating drug reimbursement prices in China [17]. Transparency reporting is essential to assess economic evaluation studies’ methods, assumptions, models, and possible biases. Decision-making for reimbursement prices of new drugs may be influenced by the information available to policy-makers derived from the economic evaluation studies’ quality and the reporting format [18, 19]. Moreover, the Chinese National Health Insurance Administration does not disclose drug price negotiating “dossiers”, including economic evaluation evidence provided by manufacturers. However, these published economic evaluations could reflect those dossiers to some extents. Previously, other studies have evaluated the quality of economic evaluation in China [20, 21]. However, the quality assessment of this economic evaluation of negotiated oncology drugs has been given little attention thus far. Therefore, the purpose of this study was to systematically review economic evaluations of negotiated oncology drugs in 2020 NRDL in mainland China [22] to assess the reporting quality of the currently available publications on this topic.

## Methods

### Literature search

A systematic review of the literature was conducted on March 31, 2021, to identify economic evaluation studies relating to negotiated oncology drugs in China’s 2020 NRDL. We used the keywords “pharmacoeconomic”, “cost-effectiveness analysis”, “cost-benefit analysis”, “cost-utility analysis”, “cost minimization analysis”, “cost analysis” and the generic name for negotiated oncology drugs on the list to search in the three English-language (PubMed/MEDLINE, Embase, and Web of Science) and three Chinese-language databases (China Knowledge Resource Integrated Database (CNKI), WanFang Database, and Chinese Biomedical Literature Database (SinoMed)). The specific search formula was combined with keywords and adjusted appropriately to different databases. In addition to database searches, the reference lists of included review studies were screened to identify additional studies that could have been missed during the systematic search.

#### Inclusion criteria

Inclusion criteria were: (1) original research on the economic evaluations of negotiated oncology drugs displayed in 2020 NRDL; (2) conducted in China; (3) manuscript published in English or Chinese.

#### Exclusion criteria

Exclusion criteria were: (1) repeatedly published and reported studies; (2) meeting abstracts, letters to the editor, expert opinion, reviews, and introduction to the pharmacoeconomic methodology; (3) studies unrelated to negotiated oncology drugs on the list or non-pharmacoeconomic; (4) studies conducted in other regions or countries.

#### Screening of studies

Two researchers independently carried out the literature search using the strategy determined in advance, then screened the studies by reading the title, abstract and full texts to identify whether the study meets inclusion and exclusion criteria. This process resolved discrepancies through consensus discussion or a third reviewer’s adjudication. Two reviewers also extracted data from full articles for methodological details, study designs, and analysis and interpretation of results into summary tables. If there were differences, they discussed them first. In the case of failure to achieve consensus, the third reviewer resolved them.

### Quality assessment

The quality of the included studies was evaluated using the 24-item Consolidated Health Economic Evaluation Reporting Standards (CHEERS) checklist [23]. the International Society of Pharmacoeconomics and Outcomes Research (ISPOR) introduced the instrument in 2013. This checklist consolidates and updates previous efforts into a single helpful reporting standard. It is not intended to prescribe how economic evaluations should be conducted, but to assess the report quality of the economic evaluation, to ensure that the methodology used is transparent and conscientious.

The CHEERS is a checklist composed of twenty-four items to evaluate six main categories: title and abstract, introduction, methods, results, discussion, and other (source of funding, and conflicts of interest) [24]. Each checklist item was scored as “1” if the study reported that item completely, “0.5” for partially reporting, “0” for not meeting the criteria, or “Not applicable” if the item did not apply to the study to estimate a summary reporting score. Due to inapplicable items, the maximum score for each study was less than or equal to 24. In addition to calculating each study’s CHEERS score, we also used a percentage of fully, partially, and not met items to transform each study’s CHEERS scores into a range of 0-100, providing us to compare the quality scores of various studies and items [25].

Two reviewers independently appraised the studies in the quality assessment process. When results differed, reviewers resolved discrepancies by discussion. If they cannot reach an agreement, the third reviewer resolves conflicts. Ethical approval was not necessary because the present study did not involve patients.

### Statistical analysis

Descriptive summary statistics were estimated for basic information and economic evaluation characteristics of included studies. The reporting quality scores’ means and standard deviation (SD) were calculated. The Shapiro–Wilk W test rejected the null hypothesis of normal distribution of CHEERS scores. We applied the non-parametric test to examine the difference in quality scores among the various characteristics. It has been shown that linear regression models are fairly robust to violations of the normality assumption [26]. Therefore, the linear regression analysis still was employed to examine the influence of basic information and economic evaluation characteristics on the reporting quality scores of included studies. All other statistical analyses were performed using Microsoft Excel 2016 and Stata version 16.0.

## Results

**Fig. 1:**
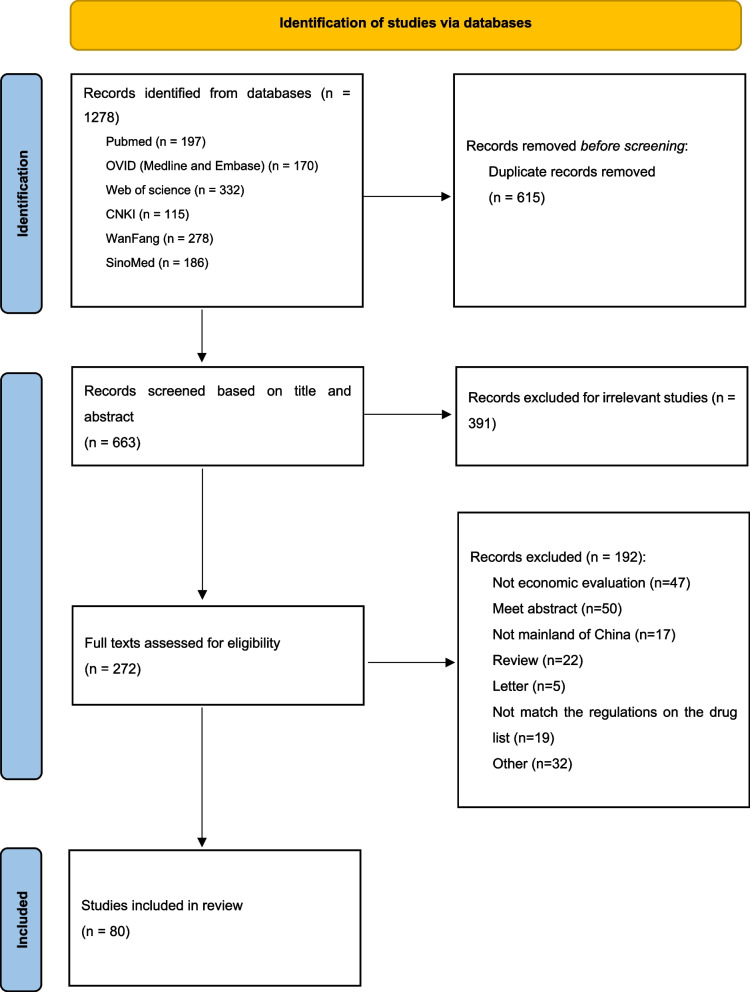
Flowchart of search results and screening process

**Fig. 2:**
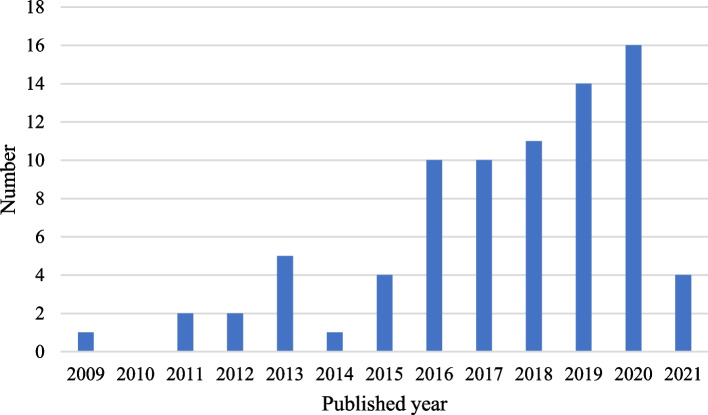
Number of articles by year

Figure 1 shows PRISMA 2020 flow diagram for literature searching. A total of 1278 records were found in the database. After removing 615 duplicates, 663 records were screened for titles and abstracts, resulting in 272 eligible studies. A full-text review revealed 192 excluded studies (not pharmacoeconomic studies, not about Mainland China, review or meeting articles, or the population using negotiated oncology drugs in the study does not match the regulations on the NRDL). Finally, 80 articles met the study’s inclusion criteria. The basic information extracted from the 80 studies [27–106] is presented in Table 1. These studies involved 20 drugs (14 targeted therapies, 5 biologics, and 1 chemotherapy) out of 42 negotiated oncology drugs. In terms of the number of authors, studies of 1 ~ 3 authors were 21 (26.25%), 4 ~ 6 authors were 36 (45.00%), and more than 6 authors were 23 (28.75%). These studies were published between 2009 and 2021. Figure 2 shows that the number of studies published has grown during this period. There have been more publications in recent years: 15 (18.75%) studies were published between 2009 and 2015, 31 (38.75%) between 2016 and 2018, and 34 (42.5%) after 2018. Most studies were published in English in international journals (46 of 80 articles, 57.5%). The affiliation of the first authors is mainly hospital (62, 77.5%), and most of the first authors (63.75%) are from the eastern region, followed by the western region (22.5%).

**Table 1:** Basic information of the included studies

Characteristic		Number	%
Author numbers	1 ~ 3	21	26.25
	4 ~ 6	36	45.00
	≥ 7	23	28.75
Published year	2009 ~ 2015	15	18.75
	2016 ~ 2018	31	38.75
	2019 ~ 2021	34	42.50
Language	English	46	57.50
	Chinese	34	42.50
Affiliation of the first author	University	18	22.50
	Hospital	62	77.50
Region of first author	the east region	51	63.75
	the central region	11	13.75
	the west region	18	22.50
Tumor type	Colorectal cancer	13	16.25
	Non-small cell lung cancer	37	46.25
	Hepatocellular carcinoma	8	10.00
	Breast cancer	7	8.75
	Gastric cancer	4	5.00
	Renal cell carcinoma	2	2.50
	Myeloid leukemia	5	6.25
	Multiple myeloma	1	1.25
	Nasopharyngeal carcinoma	1	1.25
	Ovarian Cancer	2	2.50
Method of economic evaluation	Cost-utility analysis	67	83.75
	Cost-effectiveness analysis	12	15.00
	Cost minimization analysis	1	1.25
Study design^b^	Prospective Study	4	5.00
	Retrospective Study	10	12.50
	Modeling Study	66	82.50
Source of funding	No funding	13	16.25
	Government^a^	32	40.00
	Pharmaceutical company	8	10.00
	Not mentioned	27	33.75
Study Perspective	Health insurance system	19	23.75
	Healthcare system	38	47.50
	Societal	10	12.50
	Patient	4	5.00
	Health Care Provider	1	1.25
	Not mentioned	8	10.00
Time horizon	Not mentioned	17	21.25
	< 1 year	1	1.25
	1–5 year	16	20.00
	6–10 year	31	38.75
	> 10 year	15	18.75
Total		80	100.00

^a^ Including other government-funded institutions, such as public hospitals

^b^ The economic evaluation studies based on modeling or empirical research (such as patient level or cluster level human studies). The design of this empirical study can be classified as a prospective or retrospective study. Prospective studies are studies in which the investigator conducts the study according to the requirements of the topic and design chosen and prospectively collects patient information needed for the economic evaluation. Retrospective studies tend to include patients retrospectively and collect patient information from pre-existing information such as various databases or hospital data systems

Of these studies, most of them (83.75%) conducted the cost-utility analysis, and only one article conducted the cost-minimization analysis. Most (82.5%) of studies used modeling for their analyses, and 23.75 and 47.5% were health insurance and healthcare system perspective, respectively. In addition, two-fifth (38.75%) of studies used the 6-10-year time horizon for analysis, 20% used the 1 ~ 5 year, and 18.75% used a more than 10-year time horizon. Most studies were funded by the government (40%). Non-small cell lung cancer (46.25%), colorectal cancer (16.25%), and hepatocellular carcinoma (10%) were the most common tumor type for these studies.

**Fig. 3:**
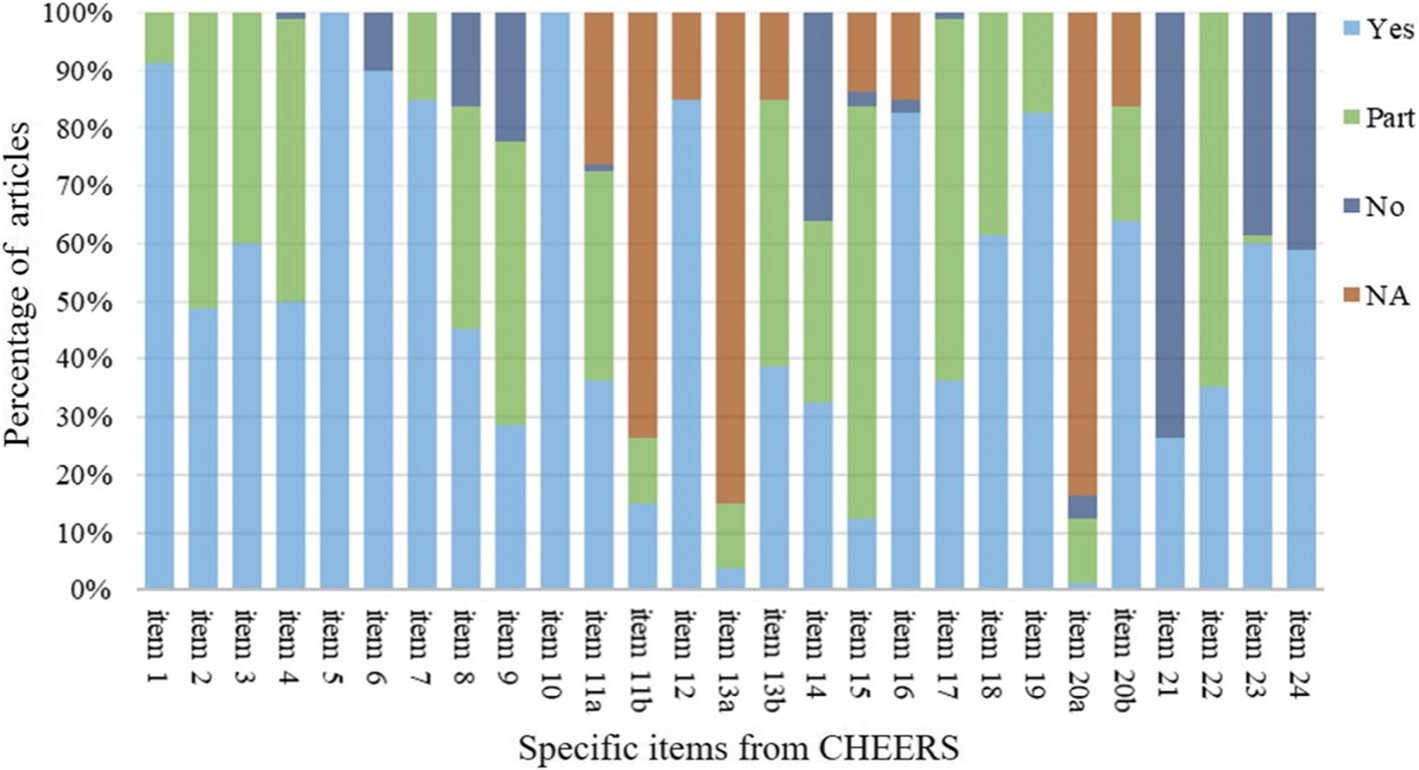
Reporting quality of publications based on per items of the CHEERS checklist

Figure 3 shows the proportion of the included studies scored as entirely adequate, partially, or not based on each CHEERS item. Several items demonstrated that less than half of the studies obtained full points, including the abstract, time horizon, discount rate, estimating resources and costs, currency, price date, and conversion, choice of model, heterogeneity, and discussion. In contrast, over 90% of the studies gave a clear title, setting and location, study perspective to estimate cost, and outcome indicators based on economic evaluation type.

Overall, the average CHEERS score of all articles was 17.68 ± 3.41 and ranged from 9.5 to 22.5 (Supplement Table 1). Converting to the 0-100 scale shown in Table 2, the average score of all articles was 74.63 ± 12.75 (range, 43.48–93.75). The average categorical scores for six main categories (title and abstract, introduction, methods, results, discussion, and other) of the CHEERS checklist were 85.00 (SD = 15.20), 80.00 (SD = 24.65), 76.97 (SD = 11.63), 69.69 (SD = 19.87), 67.50 (SD = 24.00) and 59.69 (SD = 42.93), respectively. The mean reporting score of all articles in the title and abstract was the highest (85.00), followed by the introduction section (80.00). In contrast, the other section reported the lowest mean scores, including the source of funding, and conflicts of interest items.

**Table 2:** Reporting quality scores of included studies based on CHEERS checklist

Section	N	Minimum	Maximum	Mean	SD
Title and abstract	80	50.00	100.00	85.00	15.20
Introduction	80	50.00	100.00	80.00	24.65
Methods	80	50.00	96.43	76.97	11.63
Results	80	25.00	100.00	69.69	19.87
Discussion	80	50.00	100.00	67.50	24.00
Other	80	0.00	100.00	59.69	42.93
Total	80	43.48	93.75	74.63	12.75

*CHEERS* Consolidated Health Economic Evaluation Reporting Standards, *SD* Standard deviation

Table 3 shows the CHEERS scores of all articles by the article characteristics.The Chinese articles’ scores were significantly lower than those published in English (*P* < 0.001). There was a significantly rising time trend in reporting quality scores: 68.81 (± 12.12) between 2009 and 2015), 73.69 (± 14.15) between 2016 and 2018, and 78.06 (± 10.78) after 2018 (trend testing *P*-value = 0.045). Regarding the author numbers, the articles with fewer authors assessed lower scores than those with more than six authors (*P* = 0.013). Regarding the type of economic evaluation, the mean score of articles reporting a CUA was 78.43 (± 9.27), which was significantly higher than those articles reporting a CEA and CMA (*P* < 0.001). The mean score of articles that used modeling design was 78.47 (± 9.12), significantly higher than the articles using prospective study design 49.40 (± 3.00), or the articles using retrospective study design 59.39 (± 13.14) (*p* < 0.001). The studies that used a longer time horizon for analysis had higher scores than those articles with a time horizon of less than one year (*P* < 0.001). In terms of source of funding, studies funded by the pharmaceutical industry had the highest mean scores (85.16 ± 6.34), followed by the government (79.58 ± 10.11), and studies with not mention funding sources (63.72 ± 10.55) had the lowest scores (*P* < 0.001). There are significant differences in the mean scores among articles that used different study perspectives, and the study did not mention that the study perspective had the lowest scores (*P* < 0.001).

**Table 3:** Univariate analysis of reporting quality scores for included studies

Characteristic			Mean	SD	*P* value
Author numbers	1 ~ 3		67.05	15.51	0.013
	4 ~ 6		75.98	10.93	
	≥ 7		79.46	9.62	
Published year	2009 ~ 2015		68.81	12.12	0.045
	2016 ~ 2018		73.69	14.15	
	2019 ~ 2021		78.06	10.78	
Language	English		83.51	5.80	< 0.001
	Chinese		62.62	9.20	
Affiliation of the first author	University		73.96	13.74	0.853
	Hospital		74.83	12.55	
Region of first author	the east region		74.60	13.98	0.849
	the middle region		72.10	14.30	
	the west region		76.28	7.30	
Tumor type	Colorectal cancer		77.59	12.80	0.294
	Non-small cell lung cancer		74.42	13.13	
	Hepatocellular carcinoma		80.73	8.89	
	Breast cancer		69.26	12.95	
	Gastric cancer		77.90	14.45	
	Renal cell carcinoma		69.79	1.47	
	Myeloid leukemia		73.33	12.36	
	Other		78.72	3.58	
	Ovarian Cancer		51.19	1.68	
Method of economic evaluation	Cost-utility analysis		78.43	9.27	< 0.001
	Cost-effectiveness analysis		55.88	10.04	
	Cost minimization analysis		45.24	-	
Study design	Prospective Study		49.40	3.00	< 0.001
	Retrospective Study		59.39	13.14	
	Modeling Study		78.47	9.12	
Source of funding	No funding		78.64	10.81	< 0.001
	Government^a^		79.58	10.11	
	Pharmaceutical company		85.16	6.34	
	Not mentioned		63.72	10.55	
Study Perspective	Health insurance system		75.52	10.76	< 0.001
	Healthcare system		79.86	9.14	
	Societal		72.47	12.25	
	Patient		75.14	12.44	
	Health Care Provider’s		68.75	-	
	Not mentioned		50.89	5.32	
Time horizon	Not mentioned		61.23	12.68	< 0.001
	< 1 year		45.24	-	
	1–5 year		72.34	8.32	
	6–10 year		80.38	9.31	
	> 10 year		82.36	7.08	
Total			74.63	12.75	

*SD* Standard deviation

^a^ Including other government-funded institutions, such as public hospitals

Table 4 reports the influencing factors of CHEERS scores of included studies from regression analysis. Higher scores were associated with articles published between 2019 and 2021 year (*P* < 0.05) and English publications (*P* < 0.01). Studies without the disclosed source of funding and study perspective (*P* < 0.05) were statistically significant factors of lower scores.

**Table 4:** Influencing factors of reporting quality scores from Liner regression model

Characteristic		Coefficients	SE	*P* value
Author numbers	1 ~ 3	Ref.		
	4 ~ 6	-1.555	1.665	0.354
	> 7	-1.458	1.778	0.415
Published year	2009 ~ 2015	Ref.		
	2016 ~ 2018	1.065	1.662	0.524
	2019 ~ 2021	3.678	1.643	0.029
Language	Chinese	Ref.		
	English	11.607	1.777	< 0.001
Method of economic evaluation	Cost-utility analysis	Ref.		
	Cost-effectiveness analysis	-4.165	3.506	0.240
	Cost minimization analysis	-3.540	6.747	0.602
Study design	Prospective Study	Ref.		
	Retrospective Study	-4.331	4.250	0.312
	Modeling Study	2.555	5.139	0.621
Source of funding	No funding	Ref.		
	Government^a^	0.990	1.830	0.591
	Pharmaceutical company	0.859	2.281	0.708
	Not mentioned	-4.469	2.035	0.032
Study Perspective	Health insurance system	Ref.		
	Healthcare system	0.405	1.504	0.789
	Societal	-4.639	2.002	0.024
	Patient	4.001	2.946	0.180
	Health Care Provider	-5.073	5.223	0.335
	Not mentioned	-11.173	3.435	0.002
Time horizon	Not mentioned	Ref.		
	< 1 year	-3.706	5.541	0.506
	1–5 year	1.618	2.120	0.448
	6–10 year	0.470	2.223	0.833
	> 10 year	2.427	2.454	0.327
Constant		67.686	5.808	< 0.001

*SE* Standard error

^a^ Including other government-funded institutions, such as public hospitals

## Discussion

To the best of our knowledge, this is the first systematic review to examine the reporting quality of economic evaluation studies focusing on the negotiated oncology drugs included in China’s NRDL for 2020. A drug price negotiation package should include an economic evaluation study. Transparent clear reporting and high-quality studies are essential for supporting decision-making in the process [107]. Furthermore, the Chinese National Health Insurance Administration does not disclose drug price negotiating dossiers, including economic evaluation evidence provided by manufacturers. We intended to review the currently available publications on this topic as a proxy for economic evaluation evidence from negotiations and evaluate them using CHEERS for reporting quality, and we hope to contribute to the renegotiation process in the future. The CHEERS checklist was one of the three most widely used quality assessment tools in pharmacoeconomic system review [108]. Many system reviews have used this checklist for quantitative assessment of economic evaluation since its publication [108, 109].

The overall mean score of reporting quality of economic evaluations in the present study was 17.68, and the scores ranged between 9.5 and 22.5, which showed less than 75% adherence to the CHEERS 2013 checklist. The CHEERS score was nearly the same as the reporting quality score of health economic evaluation research in India, Myanmar, Cambodia, and Laos, ranging between 17 to 17.8 [110]. Before our study, a study including pharmacoeconomic research from 2003 to 2014 in China reported a mean score of 18.7 assessed using the same CHEERS checklist, which had 1.02 higher than the score of our study [20]. The Jiehua Cheng et al. study showed that the average quality score of the included studies in China from 2006 to 2015 was 56.59 ± 16.90 [25], less than 74.63 ± 12.75 from our study. The reporting quality on China’s published economic evaluation studies of negotiated oncology drugs in 2020 latest NRDL may have been improved, but it is still lower than some studies.

The CHEERS scores can be divided into three categories: high quality for scores over 75, medium quality for scores between 50 and 75, and low quality below 50 [111, 112]. If so, the quality of included studies belongs to the medium. The most inadequately reported items included a choice of model, characterization of heterogeneity, currency, price date, and conversion, and discussion. Many studies did not describe any characterization of heterogeneity, similar to some studies [110]. Some items with lower scores were mainly incompletely reported. For example, they did not explain the reasons for the choice of the model, failed to describe the quantities of resources and unit costs precisely or adjusted the costs to the reported year. The CHEERS checklist evaluates only the reporting quality of economic evaluations, not the studies’ quality. However, some studies indicate that inadequate reporting also cannot enable readers to adequately assess the reliability of study results in supporting health decision-making [113–115]. Therefore, these items of the CHEERS should be reported in detail. For example, the drug’s cost and effectiveness measurements should be transparent and available with the data source and reasons [116, 117].

Our study showed the mean score of studies from 2019 to 2021 was higher than those of studies between 2009 and 2015. The possible reason is that China Guidelines for Pharmacoeconomic Evaluations and Manual (2015 version) were published, which further contributes to guide the implementation and reporting of economics evaluation studies. In addition, the implementation of the NRDL price negotiations in recent years in China is similar to the reimbursement of drugs in many countries [118]. It will be helpful for manufacturers to provide the published economic evaluation in the price negotiations process. Therefore, it is crucial to enhance the transparency of economic evaluation and clearly report their results for helping health policy decision-makers understanding and practically applying the economic evaluations.

There was a significant difference between the average quality scores of the studies published in Chinese and those published in English. The findings also were consistent with Jiehua Cheng et al [25]. This difference may be because the editors of Chinese journals did not require authors to report standardized economic evaluations nor to supplement their details. For Chinese publications, the authors may be required to report each part of the economic evaluation based on China Guidelines for Pharmacoeconomic Evaluations. Furthermore, the Chinese standard checklist, similar to the CHEERS, could be developed to assess the Chinese economic evaluation studies.

There were some limitations in this study. Firstly, the CHEERS was intended to qualitatively evaluate the report quality of studies without specific rules for quantitative assessment [23, 119]. We may introduce a bias against publications that are not required to follow the CHEERS guideline. Secondly, some studies were published before the publication year of the CHEERS. In addition, the updated CHHERS 2022 was not used in our study because it had not been published at the time our study was completed. Moreover, compared with CHEERS 2013, the 2022 version contains additional content related to patients or service recipients, the general public, and community or stakeholder involvement and engagement; reporting and availability of a health economic analysis plan; and the description of distributional effects, among others [120]. These studies included in the article were also largely unreported. Finally, this study only assessed the report quality of included economic evaluation studies. Although this quality does not represent the quality of economic evaluation outcomes, it is also important to the decision-making process.

## Conclusion

This study reveals moderate reporting quality of economic evaluations of negotiated oncology drugs listed in the 2020 NRDL. The number and reporting quality of economic evaluations of negotiated oncology drugs in mainland China have improved. However, most studies, especially those published in Chinese, do not fully report CHEERS items, significantly decreasing the studies’ transparency. Therefore, the reporting quality of economic evaluations conducted in mainland China should continue to improve. Also, the Chinese journals maybe explore introducing a reporting standard for economics evaluations, not only based on the CHEERS checklist.

## Supplementary Information

**Additional file 1:** **Supplementary Table 1.** The 24-item CHEERS evaluation applied on the 80 included economic evaluation studies.

## Data Availability

In this review all data are available from the referenced articles. The open access articles used for analysis during the current study are available from the corresponding author. The 24-item CHEERS evaluation applied on the 80 included economic evaluation studies is included in Supplementary material.
