# Supplementary material for: Were economic evaluations well reported for the newly listed oncology drugs in China’s national reimbursement drug list
Source: BMC Health Serv Res. 2022 Dec 3;22:1475. doi: 10.1186/s12913-022-08858-7 (PMC9719239; doi:10.1186/s12913-022-08858-7)
Supplement: Supplementary file 1 — Additional file 1: Supplementary Table 1. The 24-item CHEERS evaluation applied on the 80 included economic evaluation studies. [file 12913_2022_8858_MOESM1_ESM.docx]

**Were** **economic evaluations well reported for the** **newly listed oncology drugs in** **China’s national reimbursement drug list**

**Supplementary material**

| Table 1 the 24-item CHEERS evaluation applied on the 80 included economic evaluation studies | | | | | | | | | | | | | | | | | | | | | |
| --- | --- | --- | --- | --- | --- | --- | --- | --- | --- | --- | --- | --- | --- | --- | --- | --- | --- | --- | --- | --- | --- |
| CHEERS Item | No. | The references number of included studies | | | | | | | | | | | | | | | | | | | |
|  |  | 27 | 28 | 29 | 30 | 31 | 32 | 33 | 34 | 35 | 36 | 37 | 38 | 39 | 40 | 41 | 42 | 43 | 44 | 45 | 46 |
| Title and abstract |  |  |  |  |  |  |  |  |  |  |  |  |  |  |  |  |  |  |  |  |  |
| Title | 1 | Part | Yes | Yes | Yes | Yes | Yes | Yes | Yes | Yes | Yes | Yes | Part | Yes | Yes | Yes | Yes | Yes | Yes | Yes | Yes |
| Abstract | 2 | Yes | Part | Part | Yes | Yes | Yes | Part | Part | Part | Yes | Yes | Yes | Yes | Yes | Yes | Yes | Yes | Yes | Part | Part |
| Introduction |  |  |  |  |  |  |  |  |  |  |  |  |  |  |  |  |  |  |  |  |  |
| Background and objectives | 3 | Part | Yes | Part | Part | Part | Part | Part | Part | Part | Yes | Part | Yes | Part | Yes | Part | Part | Yes | Yes | Part | Yes |
| Methods |  |  |  |  |  |  |  |  |  |  |  |  |  |  |  |  |  |  |  |  |  |
| Target population and subgroups | 4 | Yes | Yes | Yes | Yes | Part | Yes | Yes | Yes | Part | Yes | Part | Yes | Yes | Part | Part | Yes | Part | Part | Yes | Part |
| Setting and location | 5 | Yes | Yes | Yes | Yes | Yes | Yes | Yes | Yes | Yes | Yes | Yes | Yes | Yes | Yes | Yes | Yes | Yes | Yes | Yes | Yes |
| Study perspective | 6 | Yes | No | No | Yes | Yes | Yes | Yes | Yes | No | Yes | Yes | Yes | Yes | Yes | Yes | Yes | Yes | Yes | Yes | Yes |
| Comparators | 7 | Yes | Yes | Yes | Yes | Yes | Yes | Yes | Yes | Yes | Yes | Part | Yes | Yes | Yes | Yes | Yes | Yes | Yes | Yes | Yes |
| Time horizon | 8 | No | No | No | Part | Part | Yes | Yes | Yes | Part | Yes | Yes | Part | Yes | Yes | Yes | Yes | No | Yes | Part | Yes |
| Discount rate | 9 | No | No | No | Yes | No | Part | Part | Part | Yes | Yes | Part | Part | Yes | Part | Part | Part | No | Yes | Yes | Part |
| Choice of health outcomes | 10 | Yes | Yes | Yes | Yes | Yes | Yes | Yes | Yes | Yes | Yes | Yes | Yes | Yes | Yes | Yes | Yes | Yes | Yes | Yes | Yes |
| Measurement of effectiveness | 11 |  |  |  |  |  |  |  |  |  |  |  |  |  |  |  |  |  |  |  |  |
| Single study-based estimates | 11a | NA | Part | Part | Yes | Yes | Yes | Part | Part | Yes | NA | Part | Yes | Part | Yes | Yes | Yes | Part | Part | Yes | Part |
| Synthesis-based estimates | 11b | Part | NA | NA | NA | NA | NA | NA | NA | NA | Yes | NA | NA | NA | NA | NA | NA | NA | NA | NA | NA |
| Measurement and valuation of preference-based outcomes | 12 | NA | NA | NA | Yes | Yes | Yes | Yes | Yes | Yes | Yes | Yes | Yes | Yes | Yes | Yes | Yes | NA | Yes | NA | Yes |
| Estimating resources and costs | 13 |  |  |  |  |  |  |  |  |  |  |  |  |  |  |  |  |  |  |  |  |
| Single study-based economic evaluation | 13a | Part | Part | Part | NA | NA | NA | NA | NA | NA | NA | NA | NA | NA | NA | NA | NA | Yes | NA | Part | NA |
| Model-based economic evaluation | 13b | NA | NA | NA | Part | Part | Yes | Yes | Part | Part | Part | Part | Yes | Yes | Yes | Yes | Part | NA | Yes | NA | Yes |
| Currency, price date, and conversion | 14 | No | No | No | Yes | Yes | Yes | Yes | Yes | No | Part | No | Yes | Yes | Yes | Yes | Part | Part | Part | Yes | No |
| Choice of model | 15 | NA | NA | NA | Part | Yes | Part | Part | Part | Part | Part | Yes | Part | Yes | Yes | Part | Part | NA | Part | NA | Part |
| Assumptions | 16 | NA | NA | NA | Yes | Yes | Yes | Yes | Yes | Yes | Yes | Yes | Yes | Yes | Yes | Yes | Yes | NA | Yes | NA | Yes |
| Analytical methods | 17 | Part | Part | Part | Yes | Part | Part | Part | Part | Part | Part | Part | Part | Yes | Part | Yes | Part | Part | Part | Part | Part |
| Results |  |  |  |  |  |  |  |  |  |  |  |  |  |  |  |  |  |  |  |  |  |
| Study parameters | 18 | Part | Part | Part | Yes | Part | Yes | Part | Part | Part | Yes | Part | Yes | Yes | Part | Yes | Part | Part | Yes | Part | Yes |
| Incremental costs and outcomes | 19 | Yes | Yes | Yes | Yes | Yes | Yes | Part | Yes | Part | Yes | Part | Yes | Yes | Yes | Yes | Yes | Yes | Yes | Yes | Yes |
| Characterizing uncertainty | 20 |  |  |  |  |  |  |  |  |  |  |  |  |  |  |  |  |  |  |  |  |
| Single study-based economic evaluation | 20a | Part | Part | Part | NA | NA | NA | NA | NA | NA | NA | NA | NA | NA | NA | NA | NA | Part | NA | Part | NA |
| Model-based economic evaluation | 20b | NA | NA | NA | Yes | Part | Yes | Part | Part | Part | Yes | Part | Yes | Yes | Yes | Yes | Yes | NA | Yes | NA | Yes |
| Characterizing heterogeneity | 21 | No | No | No | No | No | No | No | No | No | Yes | No | No | No | No | Yes | No | No | No | No | No |
| Discussion |  |  |  |  |  |  |  |  |  |  |  |  |  |  |  |  |  |  |  |  |  |
| Study findings, limitations, generalizability, and current knowledge | 22 | Part | Part | Part | Yes | Part | Yes | Part | Yes | Part | Part | Part | Yes | Yes | Yes | Yes | Yes | Yes | Yes | Part | Yes |
| Other |  |  |  |  |  |  |  |  |  |  |  |  |  |  |  |  |  |  |  |  |  |
| Source of funding | 23 | No | No | Yes | Yes | Yes | Yes | Yes | Yes | No | Yes | Yes | Yes | No | Yes | No | No | No | Yes | Yes | Part |
| Conflicts of interest | 24 | No | No | No | Yes | Yes | Yes | Yes | Yes | No | Yes | No | Yes | Yes | Yes | Yes | Yes | No | Yes | Yes | Yes |
| Scores |  | 11 | 10.5 | 11 | 21 | 18 | 21 | 18 | 18.5 | 13.5 | 21.5 | 15.5 | 20.5 | 21 | 21 | 21 | 18.5 | 13 | 20.5 | 16 | 18.5 |
| Scores ranged 0-100 |  | 52.38 | 50 | 52.38 | 87.5 | 75 | 87.5 | 75 | 77.08 | 56.25 | 89.58 | 64.58 | 85.42 | 87.5 | 87.5 | 87.5 | 77.08 | 61.9 | 85.42 | 76.19 | 77.08 |

| Table 1 the 24-item CHEERS evaluation applied on the 80 included economic evaluation studies (Cont.) | | | | | | | | | | | | | | | | | | | | | |
| --- | --- | --- | --- | --- | --- | --- | --- | --- | --- | --- | --- | --- | --- | --- | --- | --- | --- | --- | --- | --- | --- |
| CHEERS Item | No. | The references number of included studies | | | | | | | | | | | | | | | | | | | |
|  |  | 47 | 48 | 49 | 50 | 51 | 52 | 53 | 54 | 55 | 56 | 57 | 58 | 59 | 60 | 61 | 62 | 63 | 64 | 65 | 66 |
| Title and abstract |  |  |  |  |  |  |  |  |  |  |  |  |  |  |  |  |  |  |  |  |  |
| Title | 1 | Yes | Yes | Yes | Yes | Yes | Yes | Yes | Part | Yes | Yes | Yes | Yes | Yes | Yes | Part | Yes | Part | Yes | Part | Part |
| Abstract | 2 | Yes | Yes | Part | Part | Part | Part | Yes | Part | Yes | Yes | Part | Yes | Yes | Part | Part | Yes | Part | Part | Part | Part |
| Introduction |  |  |  |  |  |  |  |  |  |  |  |  |  |  |  |  |  |  |  |  |  |
| Background and objectives | 3 | Part | Yes | Yes | Yes | Part | Yes | Yes | Yes | Part | Yes | Yes | Yes | Yes | Yes | Part | Yes | Yes | Yes | Yes | Yes |
| Methods |  |  |  |  |  |  |  |  |  |  |  |  |  |  |  |  |  |  |  |  |  |
| Target population and subgroups | 4 | Yes | Part | Part | Yes | Yes | Yes | Part | Yes | Yes | No | Yes | Part | Part | Part | Part | Yes | Yes | Yes | Yes | Yes |
| Setting and location | 5 | Yes | Yes | Yes | Yes | Yes | Yes | Yes | Yes | Yes | Yes | Yes | Yes | Yes | Yes | Yes | Yes | Yes | Yes | Yes | Yes |
| Study perspective | 6 | Yes | Yes | Yes | Yes | No | No | Yes | Yes | Yes | Yes | Yes | Yes | Yes | Yes | Yes | Yes | Yes | Yes | No | No |
| Comparators | 7 | Yes | Yes | Yes | Yes | Part | Part | Yes | Yes | Yes | Yes | Yes | Yes | Yes | Yes | Yes | Yes | Part | Yes | Yes | Part |
| Time horizon | 8 | Yes | Part | Part | No | No | Part | Yes | Yes | Part | Part | Yes | Yes | Part | Part | No | Yes | Part | Yes | No | No |
| Discount rate | 9 | Part | Yes | Part | No | Part | No | Part | Yes | Yes | Part | No | Part | Yes | Part | No | No | Yes | No | No | No |
| Choice of health outcomes | 10 | Yes | Yes | Yes | Yes | Yes | Yes | Yes | Yes | Yes | Yes | Yes | Yes | Yes | Yes | Yes | Yes | Yes | Yes | Yes | Yes |
| Measurement of effectiveness | 11 |  |  |  |  |  |  |  |  |  |  |  |  |  |  |  |  |  |  |  |  |
| Single study-based estimates | 11a | Part | No | Yes | Yes | NA | Yes | Yes | NA | Yes | NA | NA | Yes | NA | NA | NA | Yes | Part | Yes | Yes | Yes |
| Synthesis-based estimates | 11b | NA | NA | NA | NA | Part | NA | NA | Yes | NA | Part | Yes | NA | Yes | Part | Yes | NA | NA | NA | NA | NA |
| Measurement and valuation of preference-based outcomes | 12 | Yes | Yes | Yes | NA | Yes | NA | Yes | Yes | Yes | Yes | Yes | Yes | Yes | Yes | NA | Yes | Yes | NA | NA | NA |
| Estimating resources and costs | 13 |  |  |  |  |  |  |  |  |  |  |  |  |  |  |  |  |  |  |  |  |
| Single study-based economic evaluation | 13a | NA | NA | NA | Yes | NA | Part | NA | NA | NA | NA | NA | NA | NA | NA | NA | NA | NA | Part | Part | Part |
| Model-based economic evaluation | 13b | Yes | Part | Part | NA | Part | NA | Part | Yes | Part | Part | Yes | Yes | Yes | Yes | Part | Yes | Part | NA | NA | NA |
| Currency, price date, and conversion | 14 | Yes | No | No | Part | No | No | Yes | No | No | No | Part | Part | Yes | Part | No | Part | No | Part | Part | Part |
| Choice of model | 15 | Part | Part | Yes | NA | Part | NA | Part | Part | Part | Part | Part | Part | Part | Yes | No | Yes | Part | No | NA | NA |
| Assumptions | 16 | Yes | Yes | Yes | NA | Yes | NA | Yes | Yes | Yes | Yes | Yes | Yes | Yes | Yes | No | Yes | Yes | NA | NA | NA |
| Analytical methods | 17 | Part | Part | Part | Part | Part | No | Yes | Yes | Part | Part | Part | Yes | Part | Part | Part | Part | Part | Part | Part | Part |
| Results |  |  |  |  |  |  |  |  |  |  |  |  |  |  |  |  |  |  |  |  |  |
| Study parameters | 18 | Yes | Yes | Part | Part | Part | Part | Yes | Yes | Yes | Yes | Yes | Yes | Yes | Yes | Part | Yes | Yes | Part | Part | Part |
| Incremental costs and outcomes | 19 | Yes | Yes | Yes | Part | Yes | Part | Yes | Yes | Yes | Yes | Yes | Yes | Yes | Part | Part | Yes | Part | Part | Part | Part |
| Characterizing uncertainty | 20 |  |  |  |  |  |  |  |  |  |  |  |  |  |  |  |  |  |  |  |  |
| Single study-based economic evaluation | 20a | NA | NA | NA | Part | NA | No | NA | NA | NA | NA | NA | NA | NA | NA | Part | NA | NA | No | Part | No |
| Model-based economic evaluation | 20b | Yes | Yes | Yes | NA | Yes | NA | Yes | Part | Yes | Yes | Part | Yes | Part | Yes | NA | Part | Yes | NA | NA | NA |
| Characterizing heterogeneity | 21 | No | No | No | No | No | No | No | No | No | Yes | No | No | No | No | No | Yes | No | Yes | No | No |
| Discussion |  |  |  |  |  |  |  |  |  |  |  |  |  |  |  |  |  |  |  |  |  |
| Study findings, limitations, generalizability, and current knowledge | 22 | Part | Yes | Part | Part | Yes | Part | Part | Part | Part | Part | Part | Part | Part | Part | Part | Part | Part | Part | Part | Part |
| Other |  |  |  |  |  |  |  |  |  |  |  |  |  |  |  |  |  |  |  |  |  |
| Source of funding | 23 | Yes | Yes | Yes | No | Yes | No | Yes | No | No | No | Yes | Yes | Yes | No | No | Yes | No | Yes | No | No |
| Conflicts of interest | 24 | Yes | No | No | No | No | No | Yes | No | No | No | Yes | Yes | Yes | No | No | Yes | No | No | No | No |
| Scores |  | 20 | 17.5 | 17 | 12.5 | 14.5 | 9.5 | 20.5 | 17.5 | 17 | 16.5 | 19 | 20.5 | 20 | 16.5 | 10 | 21 | 15 | 14.5 | 10.5 | 9.5 |
| Scores ranged 0-100 |  | 83.33 | 72.92 | 70.83 | 59.52 | 60.42 | 45.24 | 85.42 | 72.92 | 70.83 | 68.75 | 79.17 | 85.42 | 83.33 | 68.75 | 43.48 | 87.5 | 62.5 | 63.04 | 50 | 45.24 |

| Table 1 the 24-item CHEERS evaluation applied on the 80 included economic evaluation studies (Cont.) | | | | | | | | | | | | | | | | | | | | | |
| --- | --- | --- | --- | --- | --- | --- | --- | --- | --- | --- | --- | --- | --- | --- | --- | --- | --- | --- | --- | --- | --- |
| CHEERS Item | No. | The references number of included studies | | | | | | | | | | | | | | | | | | | |
|  |  | 67 | 68 | 69 | 70 | 71 | 72 | 73 | 74 | 75 | 76 | 77 | 78 | 79 | 80 | 81 | 82 | 83 | 84 | 85 | 86 |
| Title and abstract |  |  |  |  |  |  |  |  |  |  |  |  |  |  |  |  |  |  |  |  |  |
| Title | 1 | Yes | Yes | Yes | Yes | Yes | Yes | Yes | Yes | Yes | Yes | Yes | Yes | Yes | Yes | Yes | Yes | Yes | Yes | Yes | Yes |
| Abstract | 2 | Part | Yes | Yes | Part | Yes | Yes | Part | Part | Part | Yes | Part | Part | Part | Part | Yes | Part | Part | Part | Yes | Yes |
| Introduction |  |  |  |  |  |  |  |  |  |  |  |  |  |  |  |  |  |  |  |  |  |
| Background and objectives | 3 | Part | Yes | Yes | Yes | Yes | Yes | Yes | Yes | Yes | Yes | Yes | Yes | Yes | Part | Part | Yes | Part | Part | Part | Part |
| Methods |  |  |  |  |  |  |  |  |  |  |  |  |  |  |  |  |  |  |  |  |  |
| Target population and subgroups | 4 | Yes | Part | Part | Yes | Yes | Part | Yes | Yes | Yes | Part | Part | Part | Yes | Part | Part | Part | Part | Yes | Yes | Yes |
| Setting and location | 5 | Yes | Yes | Yes | Yes | Yes | Yes | Yes | Yes | Yes | Yes | Yes | Yes | Yes | Yes | Yes | Yes | Yes | Yes | Yes | Yes |
| Study perspective | 6 | Yes | Yes | Yes | Yes | Yes | Yes | Yes | Yes | Yes | Yes | Yes | Yes | Yes | No | Yes | Yes | Yes | Yes | Yes | Yes |
| Comparators | 7 | Yes | Part | Part | Yes | Yes | Part | Yes | Part | Part | Yes | Yes | Yes | Part | Yes | Yes | Yes | Yes | Yes | Yes | Yes |
| Time horizon | 8 | Part | Part | Yes | Yes | Yes | Yes | Part | Yes | Part | Yes | Yes | Yes | No | No | Part | Part | Part | Yes | No | Yes |
| Discount rate | 9 | Yes | Part | Part | Part | Part | Part | Part | Part | Part | Yes | Part | Part | Yes | No | Part | Part | Yes | Part | Yes | Yes |
| Choice of health outcomes | 10 | Yes | Yes | Yes | Yes | Yes | Yes | Yes | Yes | Yes | Yes | Yes | Yes | Yes | Yes | Yes | Yes | Yes | Yes | Yes | Yes |
| Measurement of effectiveness | 11 |  |  |  |  |  |  |  |  |  |  |  |  |  |  |  |  |  |  |  |  |
| Single study-based estimates | 11a | Yes | Part | Part | Part | NA | NA | Yes | Part | Part | NA | Part | Part | Yes | NA | Part | Part | Yes | NA | Part | Yes |
| Synthesis-based estimates | 11b | NA | NA | NA | NA | Yes | Yes | NA | NA | NA | Yes | NA | NA | NA | Part | NA | NA | NA | Yes | NA | NA |
| Measurement and valuation of preference-based outcomes | 12 | Yes | Yes | Yes | Yes | Yes | Yes | Yes | Yes | Yes | Yes | Yes | Yes | Yes | NA | Yes | Yes | Yes | Yes | Yes | Yes |
| Estimating resources and costs | 13 |  |  |  |  |  |  |  |  |  |  |  |  |  |  |  |  |  |  |  |  |
| Single study-based economic evaluation | 13a | NA | NA | NA | NA | NA | NA | NA | NA | NA | NA | NA | NA | Part | Yes | NA | NA | NA | NA | NA | NA |
| Model-based economic evaluation | 13b | Yes | Yes | Yes | Part | Yes | Part | Part | Part | Part | Part | Part | Yes | NA | NA | Part | Part | Yes | Yes | Part | Yes |
| Currency, price date, and conversion | 14 | No | Yes | Yes | Part | Yes | No | No | No | No | Part | Part | No | Yes | No | No | Part | Part | Part | No | Yes |
| Choice of model | 15 | Part | Part | Part | Part | Part | Part | Part | Part | Part | Part | Part | Part | NA | NA | Part | Part | Part | Part | Part | Part |
| Assumptions | 16 | Yes | Yes | Yes | Yes | Yes | Yes | Yes | Yes | Yes | Yes | Yes | Yes | NA | NA | Yes | Yes | Yes | Yes | Yes | Yes |
| Analytical methods | 17 | Yes | Yes | Part | Part | Yes | Part | Part | Yes | Yes | Yes | Part | Yes | Part | Part | Yes | Part | Yes | Yes | Yes | Yes |
| Results |  |  |  |  |  |  |  |  |  |  |  |  |  |  |  |  |  |  |  |  |  |
| Study parameters | 18 | Yes | Yes | Yes | Yes | Yes | Yes | Part | Part | Yes | Yes | Yes | Yes | Yes | Yes | Yes | Yes | Yes | Part | Part | Yes |
| Incremental costs and outcomes | 19 | Yes | Yes | Part | Yes | Yes | Yes | Yes | Yes | Yes | Yes | Yes | Yes | Yes | Part | Yes | Yes | Yes | Yes | Yes | Yes |
| Characterizing uncertainty | 20 |  |  |  |  |  |  |  |  |  |  |  |  |  |  |  |  |  |  |  |  |
| Single study-based economic evaluation | 20a | NA | NA | NA | NA | NA | NA | NA | NA | NA | NA | NA | NA | Yes | Part | NA | NA | NA | NA | NA | NA |
| Model-based economic evaluation | 20b | Yes | Yes | Yes | Part | Yes | Yes | Part | Yes | Part | Yes | Yes | Yes | NA | NA | Yes | Yes | Yes | Yes | Yes | Yes |
| Characterizing heterogeneity | 21 | Yes | Yes | Yes | No | Yes | Yes | No | No | No | No | No | No | Yes | No | No | No | No | No | Yes | No |
| Discussion |  |  |  |  |  |  |  |  |  |  |  |  |  |  |  |  |  |  |  |  |  |
| Study findings, limitations, generalizability, and current knowledge | 22 | Yes | Yes | Part | Part | Part | Yes | Part | Part | Part | Part | Part | Part | Part | Part | Part | Part | Part | Part | Yes | Yes |
| Other |  |  |  |  |  |  |  |  |  |  |  |  |  |  |  |  |  |  |  |  |  |
| Source of funding | 23 | Yes | Yes | Yes | No | Yes | No | Yes | No | Yes | Yes | Yes | No | Yes | No | Yes | No | Yes | Yes | No | Yes |
| Conflicts of interest | 24 | Yes | Yes | Yes | No | Yes | No | No | No | No | Yes | Yes | No | Yes | No | No | No | Yes | Yes | Yes | Yes |
| Scores |  | 21 | 21 | 20 | 16.5 | 22.5 | 18 | 16.5 | 16 | 16.5 | 20.5 | 18.5 | 17 | 18.5 | 10 | 17 | 16 | 19.5 | 19.5 | 18.5 | 22 |
| Scores ranged 0-100 |  | 87.5 | 87.5 | 83.33 | 68.75 | 93.75 | 75 | 68.75 | 66.67 | 68.75 | 85.42 | 77.08 | 70.83 | 84.09 | 47.62 | 70.83 | 66.67 | 81.25 | 81.25 | 77.08 | 91.67 |

| Table 1 the 24-item CHEERS evaluation applied on the 80 included economic evaluation studies (Cont.) | | | | | | | | | | | | | | | | | | | | | |
| --- | --- | --- | --- | --- | --- | --- | --- | --- | --- | --- | --- | --- | --- | --- | --- | --- | --- | --- | --- | --- | --- |
| CHEERS Item | No. | The references number of included studies | | | | | | | | | | | | | | | | | | | |
|  |  | 87 | 88 | 89 | 90 | 91 | 92 | 93 | 94 | 95 | 96 | 97 | 98 | 99 | 100 | 101 | 102 | 103 | 104 | 105 | 106 |
| Title and abstract |  |  |  |  |  |  |  |  |  |  |  |  |  |  |  |  |  |  |  |  |  |
| Title | 1 | Yes | Yes | Yes | Yes | Yes | Yes | Yes | Yes | Yes | Yes | Yes | Yes | Yes | Yes | Yes | Yes | Yes | Yes | Yes | Yes |
| Abstract | 2 | Yes | Part | Part | Part | Part | Yes | Yes | Yes | Yes | Yes | Part | Yes | Part | Part | Part | Part | Yes | Yes | Part | Yes |
| Introduction |  |  |  |  |  |  |  |  |  |  |  |  |  |  |  |  |  |  |  |  |  |
| Background and objectives | 3 | Yes | Part | Yes | Part | Yes | Part | Part | Part | Yes | Part | Yes | Yes | Yes | Yes | Yes | Yes | Yes | Part | Part | Yes |
| Methods |  |  |  |  |  |  |  |  |  |  |  |  |  |  |  |  |  |  |  |  |  |
| Target population and subgroups | 4 | Yes | Yes | Part | Part | Part | Part | Part | Yes | Part | Part | Part | Part | Part | Yes | Part | Yes | Yes | Part | Part | Part |
| Setting and location | 5 | Yes | Yes | Yes | Yes | Yes | Yes | Yes | Yes | Yes | Yes | Yes | Yes | Yes | Yes | Yes | Yes | Yes | Yes | Yes | Yes |
| Study perspective | 6 | Yes | Yes | Yes | Yes | Yes | Yes | Yes | Yes | Yes | Yes | Yes | Yes | Yes | Yes | Yes | Yes | Yes | Yes | Yes | Yes |
| Comparators | 7 | Yes | Yes | Yes | Yes | Part | Yes | Yes | Yes | Yes | Yes | Yes | Yes | Yes | Yes | Yes | Yes | Yes | Yes | Yes | Yes |
| Time horizon | 8 | No | Yes | Yes | Yes | Yes | Yes | Part | Part | Yes | Yes | Yes | Part | Part | Part | Part | Part | Part | Part | Part | Part |
| Discount rate | 9 | Part | No | Part | Part | Yes | Yes | No | Yes | No | Part | Part | Yes | Part | No | Part | Part | Part | Part | Yes | Yes |
| Choice of health outcomes | 10 | Yes | Yes | Yes | Yes | Yes | Yes | Yes | Yes | Yes | Yes | Yes | Yes | Yes | Yes | Yes | Yes | Yes | Yes | Yes | Yes |
| Measurement of effectiveness | 11 |  |  |  |  |  |  |  |  |  |  |  |  |  |  |  |  |  |  |  |  |
| Single study-based estimates | 11a | Part | Yes | NA | Part | Part | NA | Part | Yes | Part | Part | Yes | NA | NA | NA | NA | Yes | Yes | NA | Part | Part |
| Synthesis-based estimates | 11b | NA | NA | Yes | NA | NA | Yes | NA | NA | NA | NA | NA | Yes | Part | Part | Part | NA | NA | Part | NA | NA |
| Measurement and valuation of preference-based outcomes | 12 | Yes | Yes | Yes | Yes | Yes | Yes | Yes | Yes | Yes | Yes | Yes | Yes | Yes | Yes | Yes | Yes | Yes | Yes | Yes | Yes |
| Estimating resources and costs | 13 |  |  |  |  |  |  |  |  |  |  |  |  |  |  |  |  |  |  |  |  |
| Single study-based economic evaluation | 13a | NA | NA | NA | NA | NA | NA | NA | NA | NA | NA | NA | NA | NA | NA | NA | NA | NA | NA | NA | NA |
| Model-based economic evaluation | 13b | Part | Part | Part | Part | Yes | Yes | Part | Yes | Part | Yes | Part | Part | Part | Part | Part | Yes | Yes | Part | Yes | Yes |
| Currency, price date, and conversion | 14 | No | No | Yes | Yes | Yes | Yes | Yes | Yes | Part | Part | Part | Yes | No | Part | No | Part | No | Part | Part | Yes |
| Choice of model | 15 | Part | Part | Yes | Part | Part | Yes | Part | Part | Part | Yes | Part | Part | Part | Part | Part | Part | Part | Part | Part | Part |
| Assumptions | 16 | Yes | Yes | Yes | Yes | Yes | Yes | Yes | Yes | Yes | Yes | Yes | Yes | Yes | Yes | No | Yes | Yes | Yes | Yes | Yes |
| Analytical methods | 17 | Part | Part | Yes | Yes | Yes | Yes | Part | Yes | Yes | Yes | Yes | Yes | Part | Part | Part | Part | Part | Yes | Part | Yes |
| Results |  |  |  |  |  |  |  |  |  |  |  |  |  |  |  |  |  |  |  |  |  |
| Study parameters | 18 | Yes | Part | Yes | Part | Part | Yes | Part | Yes | Yes | Yes | Yes | Part | Yes | Part | Part | Yes | Yes | Yes | Yes | Yes |
| Incremental costs and outcomes | 19 | Yes | Yes | Yes | Yes | Yes | Yes | Yes | Yes | Yes | Yes | Yes | Yes | Yes | Yes | Yes | Yes | Yes | Part | Yes | Yes |
| Characterizing uncertainty | 20 |  |  |  |  |  |  |  |  |  |  |  |  |  |  |  |  |  |  |  |  |
| Single study-based economic evaluation | 20a | NA | NA | NA | NA | NA | NA | NA | NA | NA | NA | NA | NA | NA | NA | NA | NA | NA | NA | NA | NA |
| Model-based economic evaluation | 20b | Yes | Yes | Yes | Yes | Part | Yes | Yes | Yes | Yes | Yes | Yes | Yes | Yes | Yes | Part | Part | Yes | Yes | Part | Yes |
| Characterizing heterogeneity | 21 | Yes | No | Yes | No | No | No | Yes | Yes | No | Yes | Yes | No | No | No | No | No | No | Yes | Yes | Yes |
| Discussion |  |  |  |  |  |  |  |  |  |  |  |  |  |  |  |  |  |  |  |  |  |
| Study findings, limitations, generalizability, and current knowledge | 22 | Yes | Part | Yes | Part | Part | Part | Yes | Yes | Part | Yes | Yes | Yes | Part | Part | Part | Yes | Yes | Part | Part | Yes |
| Other |  |  |  |  |  |  |  |  |  |  |  |  |  |  |  |  |  |  |  |  |  |
| Source of funding | 23 | No | No | Yes | No | No | Yes | No | Yes | Yes | Yes | Yes | Yes | No | No | Yes | Yes | Yes | Yes | Yes | Yes |
| Conflicts of interest | 24 | Yes | Yes | Yes | No | No | Yes | Yes | Yes | Yes | Yes | Yes | Yes | No | Yes | No | Yes | Yes | Yes | Yes | Yes |
| Scores |  | 18.5 | 16.5 | 22 | 16.5 | 17 | 21.5 | 18 | 22.5 | 19 | 21.5 | 21 | 20.5 | 15.5 | 16.5 | 14.5 | 19.5 | 20 | 19 | 19 | 22 |
| Scores ranged 0-100 |  | 77.08 | 68.75 | 91.67 | 68.75 | 70.83 | 89.58 | 75 | 93.75 | 79.17 | 89.58 | 87.5 | 85.42 | 64.58 | 68.75 | 60.42 | 81.25 | 83.33 | 79.17 | 79.17 | 91.67 |

Supplement Table 1 the 24-item CHEERS evaluation applied on the 80 included economic evaluation studies
